# The foreign language effect on the self-serving bias: A field experiment in the high school classroom

**DOI:** 10.1371/journal.pone.0192143

**Published:** 2018-02-09

**Authors:** Joeri van Hugten, Arjen van Witteloostuijn

**Affiliations:** 1 Tilburg School of Economics and Management, Tilburg University, Tilburg, the Netherlands; 2 School of Business and Economics, Free University Amsterdam, Amsterdam, the Netherlands; 3 Antwerp School of Management, University of Antwerp, Antwerp, Belgium; Kyoto University, JAPAN

## Abstract

The rise of bilingual education triggers an important question: which language is preferred for a particular school activity? Our field experiment (*n* = 120) shows that students (aged 13–15) who process feedback in non-native English have greater self-serving bias than students who process feedback in their native Dutch. By contrast, literature on the foreign-language emotionality effect suggests a weaker self-serving bias in the non-native language, so our result adds nuance to that literature. The result is important to schools as it suggests that teachers may be able to reduce students’ defensiveness and demotivation by communicating negative feedback in the native language, and teachers may be able to increase students’ confidence and motivation by communicating positive feedback in the foreign language.

## Introduction

Schools increasingly have a choice between using a more cosmopolitan language (e.g., English in Western countries) or using students’ native language in the classroom. The consequences of this choice are poorly understood, but they appear to be important. For instance, the language of a public goods game influences the likelihood to free ride [1], the language of a trolley dilemma impacts the likelihood of sacrificing one person to save five [2], and the language of a prisoner’s dilemma game affects the likelihood of choosing to cooperate [3]. Using a foreign language also reduces several decision-making biases [4,5], an example being loss aversion [6].

In this paper, we focus on a new and important consequence: the information-processing bias called the ‘self-serving bias’ [7,8]. A self-serving bias means that individuals tend to attribute positive feedback to their own ability, while they attribute negative feedback to external factors. The self-serving bias plays an important role in self-esteem and motivation [9]. Our research question is: Does the language in which feedback is processed–i.e. native (Dutch) versus non-native (English)–influence the self-serving bias? We conduct a field experiment in which we ask early teenagers doing bilingual (Dutch and English) education to what extent they attribute to their own ability their performance on a puzzle task in the classroom. We randomly assign students to receive positive or negative feedback from the task. Furthermore, we randomly assign English versus native language feedback, and ask the students, in that language, to make an attribution. We report how the difference in attribution between positive and negative feedback conditions differs between the Dutch and English conditions.

The theoretical lens that we use involves the foreign-language effect [1,4,5,10–12]. That lens views foreign language as distant from emotions, whilst native language and emotions are intertwined. We do not invoke dual process theory [13], like related work does [1,5], because the more modest concept of arousal is sufficient for us to make predictions, but our study is consistent with that work. The lens is potentially useful because it highlights emotion as a potential mechanism by which the choice of language could influence self-serving bias. Specifically, feedback in a foreign language may be processed less emotionally, which may reduce the self-serving bias.

Our study has several contributions. First, it advances research on the foreign language effect by focusing on a new consequence: self-serving bias. The most closely related consequence in the existing literature on the foreign language effect is loss aversion [6]. Second, our study adds nuance to existing research on the foreign language effect by including a potential moderator: foreign language anxiety. Existing literature has only used foreign language anxiety as an explanatory mechanism [10] or outcome [12]. Third, we propose a new factor that can influence the strength of the self-serving bias: whether the feedback is in the native or foreign language. The most closely related antecedent in the existing literature on the self-serving bias is culture [8]. Furthermore, we conduct a field experiment in a Dutch high school setting, with 120 students aged 13–15. Our field experiment is one of the first, to the best of our knowledge, to empirically test a consequence of the foreign language effect that is relevant to the classroom, and test it in a real classroom setting.

## Hypotheses

### The self-serving bias

We focus on one aspect of the attribution process: the self-serving bias. The self-serving bias means that individuals tend to protect their self-concept from threats by attributing negative feedback to external causes (e.g., the task was difficult, or colleagues were distracting), whilst attributing positive feedback to internal causes (e.g., I have high ability, or I can really focus well) [7]. For example, nobody attributes their good performance to the un-distractingness of their colleagues. Meta-analyses show a powerful self-serving bias, on average, but one of the weakest self-serving biases for samples of Western Europeans (non-UK) and early teenagers (i.e., our empirical context) [8]. Thus, it is still doubtful whether we can replicate this well-established main effect in our sample.

Hypothesis 1 (H1): Students have a self-serving bias such that they attribute their performance to internal factors (i.e., ability) more after receiving positive feedback than after receiving negative feedback.

### The foreign language effect

The foreign language effect refers to a difference between languages in an outcome caused by differences in emotionality of the native versus foreign language. The self-serving bias may be such an outcome. The first logical step in our argument is that foreign language has been found to reduce arousal, measured as skin conductance response [10,14] and through self-reported emotional intensity ratings [12,15].

The extent to which foreign language reduces arousal depends on several factors. First, the type of communication matters. The language in which people experience being reprimanded has a strong effect on arousal, but the language in which people tell lies makes little difference for arousal [10,15]. Possibly, the negative feedback in our empirical context is experienced like a reprimand, such that language has a strong effect. Alternatively, engaging in finding self-serving excuses for low performance may be experienced like lying, where language has a weak effect [16,17]. Second, the foreign language effect tends to be weaker for early learner of the foreign language [12,15]. In our empirical context, the participants are 13 to 15 years old, so they are all early learners. In sum, although it is not certain in our context, our baseline premise is that the foreign language is associated with lower arousal than the native language.

The next logical step in our argument is that arousal (also measured as skin conductance response) is associated with greater self-serving bias [18,19]. Theoretically, arousal reflects both (1) the negative psychophysiological state that individuals are driven to alleviate by attributing negative feedback to external factors [7], and (2) the positive psychophysiological state that individuals seek to maintain by attributing positive feedback to themselves. Thus, in theory, arousal is a mediator between feedback and attribution. Empirically, prior studies capture the role of arousal by framing it as a moderator of the effect of feedback valence on the self-directedness of attribution. Arousal positively moderates the self-serving bias for both naturally-induced arousal (i.e., arousal because of the feedback) [18], as well as experimentally-induced arousal (e.g., physical exercise just before getting feedback) [19].

Hypothesis 2 (H2): Students who make attributions in a foreign language have less self-serving bias than students who make attributions in their native language.

### Foreign language anxiety

Foreign language anxiety (FLA) is the tension and nervousness (associated with arousal of the autonomic nervous system) that are responses to the challenge to an individual’s self-concept as a competent communicator by the stimulus of being asked to use a foreign language. It consistently moderates the effect of foreign language learning efforts on performance [20]. FLA may also moderate a variety of foreign language effects [12]. FLA’s definition suggests an association with the mechanism in H2: arousal. Communicating in a foreign language requires more mental effort, which can manifest as arousal. For people with low ability in the foreign language, communicating in the foreign language may induce anxiety about making mistakes or making a bad impression, which leads to arousal [20]. So, the arousal-reducing effect of foreign language may be counteracted by such effort and anxiety [10].

Simcox, Pilotti, Mahamane, and Romero [21] find such counteracting effects. The foreign language dampens the impact on arousal of negative (e.g., taboo) versus emotionally-neutral words, but that dampening is outweighed by an arousal-increasing effect that is independent of the emotional intensity of the stimulus. By contrast, Eilola and Havelka [14] report consistent effects. The foreign language decreases arousal overall, and reduces the difference in arousal between negative (e.g., taboo) and emotionally-neutral words. We expect the counteracting effect to be especially likely to dominate for students with high FLA. Thus, we isolate the arousal-increasing effect of foreign language in the interaction with FLA.

Hypothesis 3 (H3): For students with high foreign language anxiety, the difference in self-serving bias between foreign versus native language is smaller or reversed.

## Data and methods

### The field

To collect data, we ran a field experiment. A laboratory experiment is less suitable for our purposes because the sterile laboratory setting may preclude some attributions that would substitute for ability in a realistic setting. Exactly the distractions by other people in the room or outside the window that happen in a classroom are the factors to which people may biasedly attribute bad performance. With this study, we wanted to explore the boundary condition of age for the language effect. Most studies are on college-aged people. We aim to see if the language effect also occurs in younger people (i.e., 13- to 15-year-olds). To stay close to reality, we need a setting where those people regularly receive serious feedback. We also need to have participants with enough familiarity with a foreign language to be able to process feedback in that language. Finally, we want the foreign language to be English because that is the most common option available as a foreign language, thus making the results more generalizable and comparable across studies.

Dutch high schools with bilingual education meet all those criteria. Students doing bilingual education get about half of their courses taught in English (e.g., math and physical education) and half in Dutch. The program starts at age 12 after students finish elementary school, and lasts until 17 or 18. After the third school year, classes switch to Dutch for courses for which there is a national exam (standardized to be in Dutch) at the end of the fifth or sixth year. To compensate, additional English literature classes are added, which allow students to achieve a certificate for excellent English skill. Students in bilingual education are optimally prepared for an English-language follow-up study and international work environment later in life. We focus on second-year and third-year classes.

We contacted the bilingual education coordinators of eight high schools providing bilingual education that were geographically closest to the university, located in a medium-sized city in the south of the Netherlands. We explained the purpose of our study and that we were looking for students to do a task, so that we could compare the effect of a low versus a high score between Dutch and English conditions. Two coordinators replied that they were interested in the study, and each consented to participate. We did not ask students for their individual consent. Each coordinator arranged the participation of all classes in their respective schools that had bilingual education and which had students within our target age range, for a grand total of 140 students. Our study has a 2x2 between-subject design. Applying the rule-of-thumb threshold of 30 subjects per cell, we required 120 students. Thus, after achieving that target sample size with the two schools, we stopped our search.

### Experimental design

The two schools offered the first author the opportunity to take over a regular class for a total of seven groups of students. The regular teacher was also in the classroom. At the beginning of those lectures, the first author announced that the class was going to do an experiment. That introduction only told the students to read carefully and that everything would be explained afterwards. The up-front information was intentionally sparse to keep the natural context intact, and to run the study as a field experiment rather than as a laboratory experiment in a classroom. Then, the students started the task on their phone, tablet, or computer. Students use such devices all the time for regular classroom activities. Subjects were randomly assigned to a webpage that asked for their name and explained the task in Dutch or English. Asking for names might help a subject’s self to be involved enough to trigger a self-serving bias [7]. It is also something that would be asked if students take an exam in the natural classroom setting. The task was to rearrange alphabetically ordered letters into the order in which they form a word (e.g., aloppt = laptop). The words themselves were identical in both languages to prevent language proficiency from affecting the performance, while allowing for subjects to attribute their performance to language proficiency.

One issue with this setup is that the subjects in the Dutch condition may make attributions to their general language ability, puzzle ability, or general cognitive ability, whereas English-specific language ability may be the salient ability to make attributions to for subjects in the English condition. That subject’s assigned language correlates with the kind of ability they make attributions to is some unfortunate noise. To potentially address such noise, we considered using a language-neutral task, but ultimately decided against this option because (1) potential language-neutral tasks might be mathematical, and students have strong existing beliefs about their math ability, which means that it may only be weakly influenced by the feedback from the experiment; (2) other potential language-neutral tasks might be too trivial for students to engage with seriously; and (3) having one clear language that is the same between conditions may lead to a jarring language switch between the task and the feedback.

Subjects were randomly assigned to a difficult or easy version of the task, such that they would be, by extension, assigned to realistic negative or positive feedback, respectively. So, the randomization of feedback valence is indirect. Lab experiments may prefer a more direct randomization of feedback by making it independent of performance in the task, and then obscure the right or wrong answers, or deceive subject about the performance of peers, to avoid subject’s suspicion [22]. We chose veridical feedback without obscuring or deceiving such that we more closely resembled the natural classroom setting. The difficulty level was solely determined by the length of the words to be formed: six- or seven-letter words for the difficult version, and five or six letters for the easy version. The words were emotionally neutral to avoid priming arousal or self-serving bias in other ways than by the feedback’s valence and language (e.g., [23]). Subjects did not know which version they received. They did not know that there were different versions, but some did notice differences with their neighbors. Pretests revealed that getting 30 seconds to solve each word is appropriate for early teenagers, but short enough to leave open the possibility to blame time instead of ability.

After the task, subjects received one line of neutral objective feedback with their true score: e.g., “Your score on the task is 5 out of 20” in Dutch or English. Such bare-bones feedback should be a powerful cue for the student’s internal attribution process without imposing an attribution or emotion on the student. By contrast, other studies augment the feedback by explicitly referring to ‘success’ or ‘failure’ or by adding bogus comparisons such as ‘you scored in the top 15% of all students at your university’ (e.g., [18,23,24]). We avoid such augmentations because they do not fit a natural classroom setting. The neutral nature of our feedback format caused some subjects to express (in their answer to the open attribution question) disappointment even if they scored higher than average. In other words, they were in the positive feedback condition, but we allowed them to experience the feedback as not positive. This confound is an argument for how a lab experiment could provide more accurate scientific knowledge about the direct effect between concepts. On the other hand, it is also an argument for how such accurate scientific knowledge would ultimately not help teachers decide about a noisy effect between behaviors. Our field experiment makes this trade-off in favor of such practical knowledge.

Subsequently, subjects in the English condition got English questions about attributing a cause to their performance. The first is an open question: “Why do you think you achieved that score?” The subjects had some space to write an answer of at least four words. That should immerse them, and their attribution process, in the English language. Then, we gave four Likert scales to indicate the extent to which they attribute performance to ability, task difficulty, effort, and luck. Specifically, we asked: “Think about the reason or reasons you have written above. How much do you agree with the following?” Students then had to score all four attributions. For instance, “My score:—is not because of my ability” to, at the other end of the scale, “- is because of my high or low ability.” Subjects in the Dutch condition got those questions in Dutch, such that their attribution process should be immersed in, or take place in the context of, the Dutch language. Measuring attribution dimensionally (e.g., how internal is the cause, or how stable is the cause) tends to give more powerful self-serving biases [8], but we deemed the dimensional questions too difficult for our subjects. Subjects could go back and forth between the Likert scales and the open question.

In sum, we have a 2x2 between-subject design [difficulty x language]. Leaving the moderating effect of FLA aside for now, our design is suitable to test Hypotheses 1 and 2. To investigate Hypothesis 1, subjects in the difficult condition received negative feedback, predicting that they would attribute their performance less to their ability as per the self-serving bias. To examine Hypothesis 2, subjects in the difficult x English condition received negative feedback, and are expected to process it in a less emotional way such that they would attribute their performance to their ability more than subjects in the difficult x Dutch condition, but less so than subjects in the easy conditions.

Additionally, to explore Hypothesis 3, we administered a short foreign language anxiety (FLA) scale [25]. The scale is administered in English, also to the subjects in the Dutch conditions. To control for whether the scale systematically influenced emotions during the feedback processing (e.g., as a prime [23]), and to prevent that the feedback systematically influenced how subjects rated themselves on the scale, subjects randomly received the FLA scale before the task or after all other questions. Finally, we added some additional questions about the subject’s age of acquisition of (i.e., first started learning the English language, with the four categories “1 = from birth”, “2 = from 2–5 years old”, “3 = 6–10 years old”, and “4 = eleven years old or older”), languages used at home, and gender. The foreign language effect may be stronger for students for whom English is not a second language but a third language [26]. So, the hypotheses should hold for them as well, if not more so. Thus, we include them in our analyses.

After the questions, the students waited in their seats for everyone in class to finish. Then, an interactive lecture about the experiment served as debriefing. For instance, the students talked about how they experienced the treatment. Some students were confused about the task, due to not reading the explanation carefully. Some students recognized that the words were the same in both languages, and others felt they had completed the task in Dutch or English. Some students could not recall in which language they answered the evaluation questions. Some students answered in English even if a question was posed in Dutch. Possibly, this is because the language of the guest lecture was English. To questions about thinking differently in different languages, students’ answers reflected FLA and skill; it was difficult for them to articulate whether they experienced emotions differently in different languages or whether they process information intuitively versus deliberately. Special care was taken to explain that students should not feel bad for getting a low score, or a lower score than their classmate, because the task was designed to make them get a low score, especially in the difficult condition.

### Statistical analysis

To test H1-3, we use plots of the distribution of attribution to ability, effort, task difficulty, and luck for each condition. To test H1, we check if the histogram of attribution to ability has more high values in the easy conditions than in the difficult conditions. An alternative test of H1 is to look at the histograms of internal attribution (i.e., ability and effort) minus external attributions (i.e., task difficulty and luck). In their meta-analysis, Campbell and Sedikides [7] subtract external attributions for 46 experiments, and use internal attribution only for 23 experiments (because those studies did have data on external attributions). Mezulis et al.’s [8] meta-analysis uses only ability attributions instead of internal attributions (i.e., ability and effort). They argue that effort is an unstable characteristic, implying that it does not harm the self-concept to attribute negative feedback to it, so that it is not self-serving to avoid attributing to effort. Furthermore, they do not subtract external attributions from the ability attribution. However, they also include source studies with dimensional measures (e.g., how internal is the cause, and how stable is the cause?). Thus, that kind of measurement has subtraction implicitly. Indeed, they turn out to result in a slightly greater self-serving bias. We agree with Mezulis et al.’s argument, and will therefore measure the self-serving bias as the attribution to ability rather than effort. To be consistent, subtracting external attributions should be adjusted to subtract all non-ability attributions (i.e., including effort).

To test H2, we check if those histograms have most high values in the easy conditions, a moderate amount of high values in the difficult x English condition, and the fewest in the difficult x Dutch condition. In other words, the difference between the histograms for easy x English and difficult x English is smaller than the difference between the histograms for easy x Dutch and difficult x Dutch. Because all those conditions are randomly assigned, it is not necessary to have control variables included, yet. However, to give a sense of statistical significance, we also run regression models with the difficulty and language variables, and FLA as a triple interaction, and with control variables for gender, English grade, and a dummy for whether a student was in the 2^nd^ or 3^rd^ year, because FLA is not randomly assigned. Gender captures personality differences that are correlated with different levels of FLA [25] and with different levels of self-serving bias [7]. Students with higher English grades may have lower FLA and may be more confident in attributing their performance to their English ability [7]. Finally, 3^rd^-year students have had more exposure to the foreign language, so they may have lower FLA, while their age may correlate with self-serving bias [8].

Experimental designs often involve manipulation checks. In our case, for example, we can check whether the subjects in the difficult condition indeed received more negative feedback. A manipulation check is that the average score in the difficult condition is lower than in the easy condition, implying the manipulation was successful. In addition to that manipulation check, we run a model with score as the feedback-valence variable instead of the dummy indicator of the condition,. We also do such doublechecks with the two other central variables. The language in which feedback is processed is manipulated by the language of the feedback and evaluation questions. A manipulation check is that most of the subjects in the Dutch condition answered in Dutch, and few subjects in the English condition answered in Dutch, and that therefore the manipulation was mostly successful. To doublecheck, we run models with answer language as the language variable instead of question language. For the dependent variable, attribution is measured using a Likert scale. A manipulation check is to verify if the response to the scale is in line with the answer to the open question. To doublecheck, we also code attribution in the open question, and run models on that variable instead. The robustness checks change the nature of those variables. For instance, the dependent variable becomes dichotomous. Thus, our method of analysis changes accordingly: a histogram is not informative over a regression coefficient; the model must be nonlinear instead of OLS.

### Covariates

We have many expectations about relationships between our variables. Those expectations that are relevant to the contribution of this paper are stated as hypotheses. Other expectations are interesting as replications of prior studies. We list those here. First, Hodgins et al. [23] find that a control-orientation prime (by making sentences with words such as ‘expectation’ and ‘forced’) increases self-serving bias as opposed to an autonomy-orientation prime. The FLA scale may also act as a prime for the control orientation. Subjects who complete the FLA scale before the feedback evaluation may be primed to react more anxiously, and thus have greater self-serving bias than subjects who complete the FLA scale after the feedback evaluation. Second, in their meta-analysis of self-serving bias, Campbell and Sedikides [7] find that men have greater self-serving bias than women. We expect to replicate that pattern. Third, furthermore, they find that individuals with greater self-esteem have greater self-serving bias. Possibly, students with higher English grades have greater self-esteem with respect to the experimental task. Thus, we expect that students with higher English grades have greater self-serving bias.

## Results

Some participants misunderstood the task, answering nothing, or answering “yes the letters are in alphabetical order”. They indicated their misunderstanding in the open attribution question instead of giving an attribution for their score, and some of them filled in the middle of the Likert scale for all questions. Thus, we exclude them. The sample includes seven native English speakers. Seven observations are not enough to do a subsample analysis to check whether the foreign language effect also holds from native English to non-native Dutch. Thus, we exclude them. Hence, the final sample size is *n* = 120. Data of the unreduced sample are in the supporting information files. The manipulation checks are supportive. That is, there is little overlap in score between the easy and difficult condition, and participants in the English condition rarely use Dutch as their operational language and vice-versa. (see S2 Fig).

**Fig 1:**
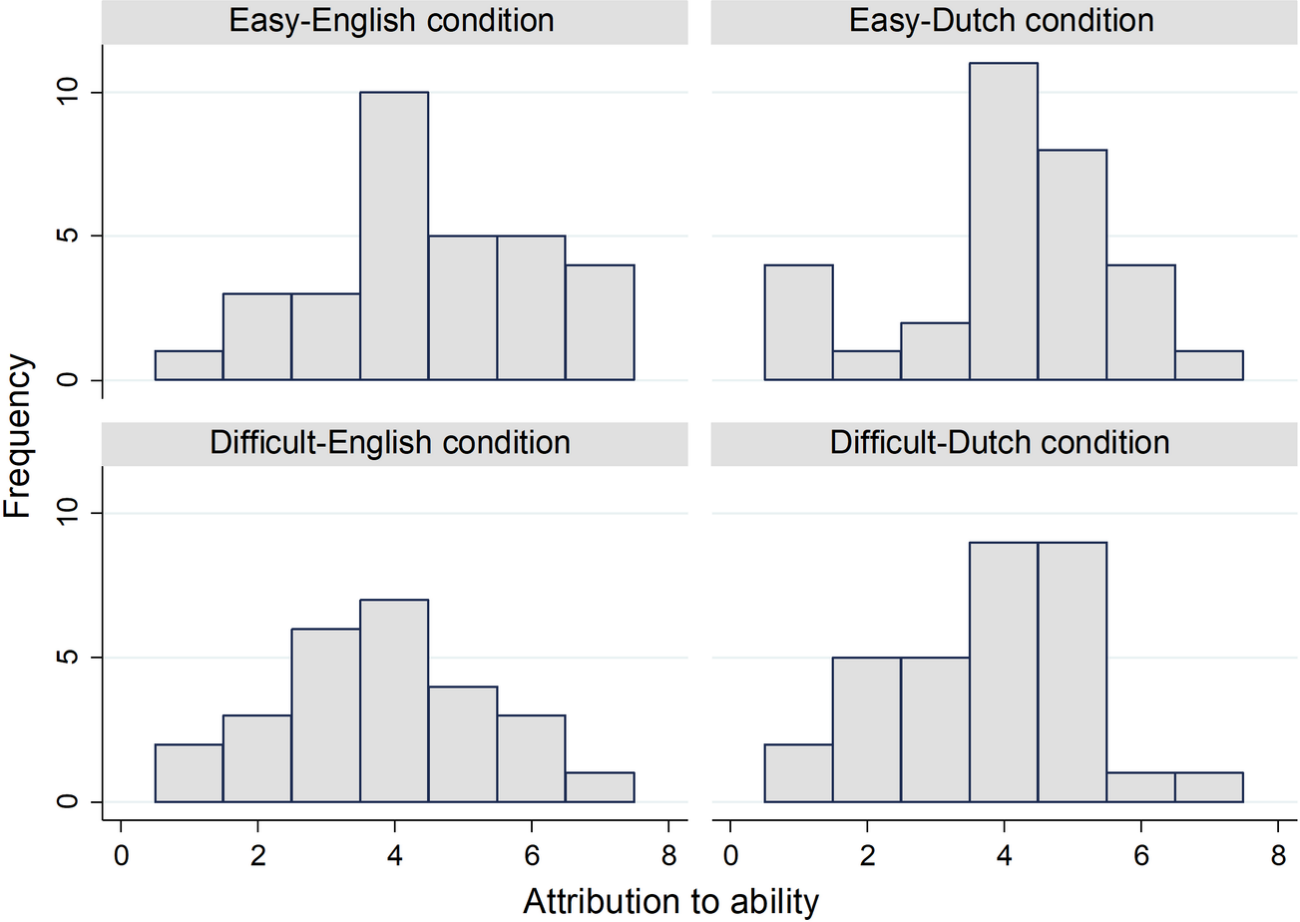
Histograms of attribution to ability. *n* = 120. By condition: *n* = 31, *n* = 31, *n* = 26 (Difficult-English), and *n* = 32 (Difficult-Dutch).

Table 1 is supportive of H1: The average attribution to ability is greater in the easy versions. The difference in Table 1 between the easy and difficult conditions for both languages, divided by the respective standard deviations, is about 0.41. By comparison, the average effect size in prior studies is 0.47 [7], or 0.7 for Western countries [8]. Our slightly smaller effect size may reflect the age and culture of our sample [8]. Fig 1 shows a histogram of attribution to ability for each condition.

**Table 1:** Mean and standard deviation of ability attributions by difficulty and language.

	English	Dutch
Easy	4.48 (1.61)	4.10 (1.58)
Difficult	3.81 (1.55)	3.78 (1.43)
Self-serving bias	0.67	0.32

**Fig 2:**
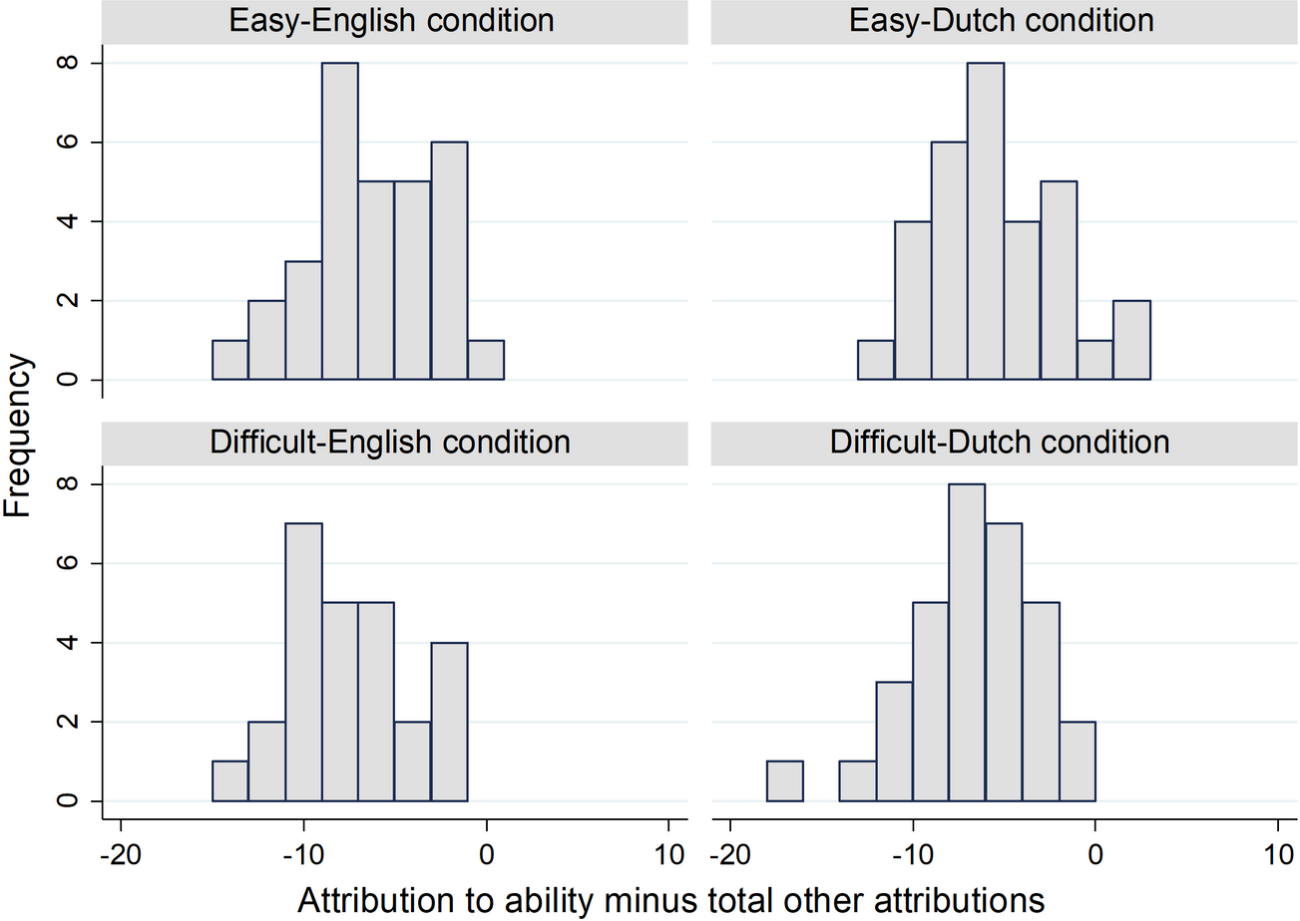
Histograms of attribution to ability minus total other.

In Table 1, attribution to ability in the easy-English condition is so great that the difference between easy-English and difficult-English conditions is greater than the difference between easy-Dutch and difficult-Dutch. That is against H2. Our methods and theory do not specify whether we should subtract the raw attributions to luck, task difficulty and effort, or whether we should subtract the average of those attributions. Fig 2 and Tables 2 and 3 compare those alternatives. None of the three options supports H2. Fig 1 suggests that the many 1s in the easy-Dutch condition lead the mean to be closer to the difficult-Dutch condition mean. Furthermore, the many 7s in the easy-English condition imply that the mean is distant from the difficult-English mean.

**Table 2:** Mean and standard deviation of attribution to ability minus the average of the luck, task difficulty, and effort attributions, by difficulty and language.

	English	Dutch
Easy	0.81 (1.72)	0.80 (1.64)
Difficult	-0.15 (1.86)	0.06 (1.91)
Self-serving bias	0.96	0.74

**Table 3:** Mean and standard deviation of attribution to ability minus total luck, task difficulty, and effort attributions, by difficulty and language.

	English	Dutch
Easy	-6.55 (3.43)	-5.81 (3.45)
Difficult	-8.08 (3.44)	-7.38 (3.71)
Self-serving bias	1.53	1.57

We can evaluate H2’s statistical significance after including foreign language anxiety (FLA) as a moderator. The analysis with FLA also includes control variables for gender, current average English grade, and whether the student is in the 2^nd^ or 3^rd^ year. These variables are not perfectly equally distributed over the experimental conditions. Females make up 65% of participants in the difficult conditions, but only 55% in the easy-English condition; 3^rd^-year students make up 47% of participants in the difficult-Dutch condition, but only 35% in the difficult-English condition. Average FLA does not differ between the conditions. The average English grade is about 7 for all conditions, except for the difficult-Dutch condition with 7.4.

**Fig 3:**
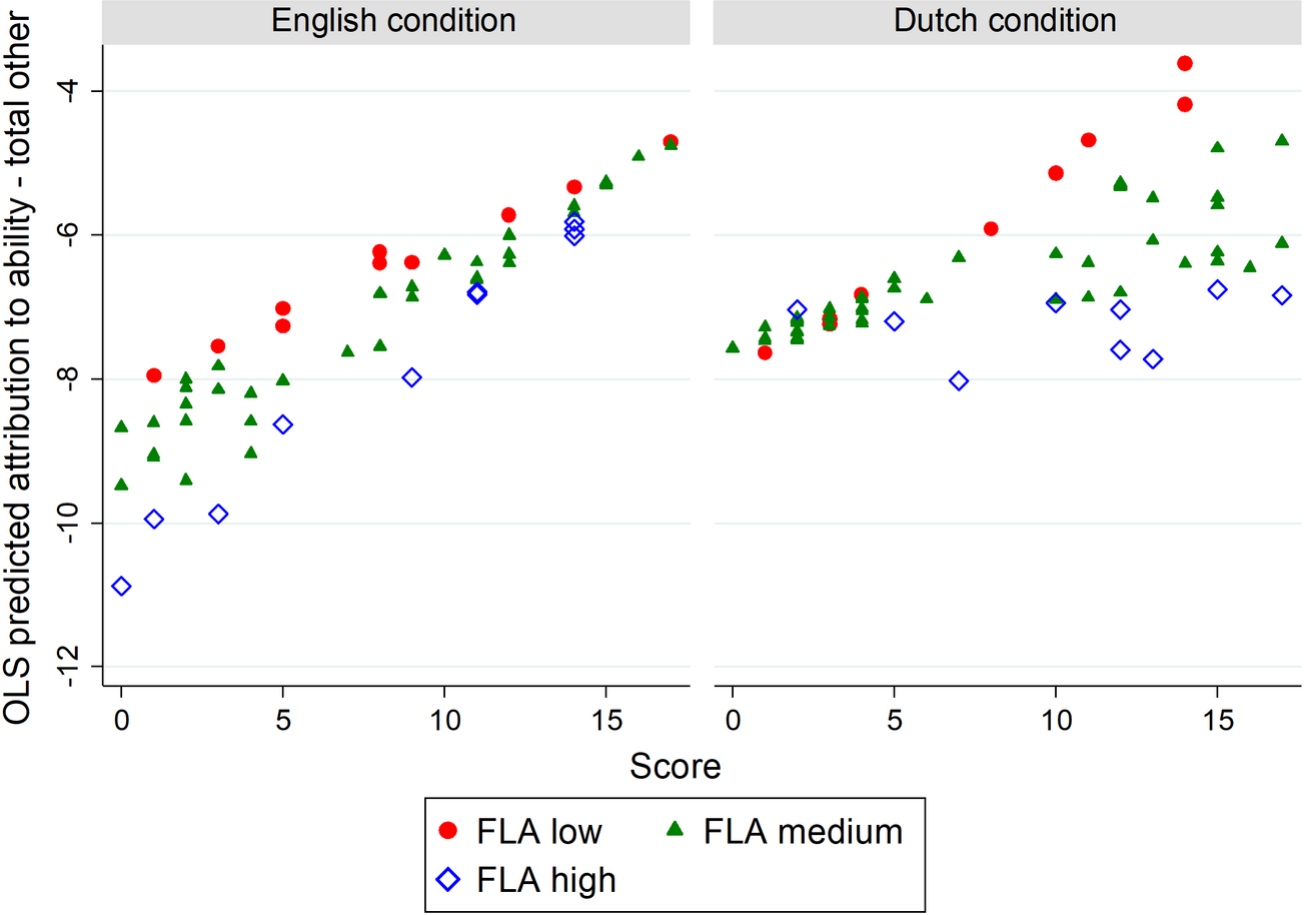
OLS analysis on attribution to ability minus total other attributions. Fig 3 is based on a model regressing attribution to ability on score achieved, language condition, the interaction between them, and a continuous measure of FLA and its interaction with score and language condition separately and combined, and control variables for gender, current average English grade, and whether the participant was in the 2^nd^ or 3^rd^ year.

Fig 3 presents the main results for H2 and H3 in graph form, for one specification. Various other specifications and regression coefficients are shown in S1 Table. For illustrative purposes, we divide the continuous variable FLA into three groups (high-medium-low). We define high FLA as an average of 4 out of 7 or greater on the FLA scale. Only 22 participants have high FLA (the median FLA is 2.9). We define low FLA as an average of 2 out of 7 or less. Furthermore, we use the ratio variable ‘score’ instead of the categorical variable ‘difficulty’.

In Fig 3, the result is opposed to H2: The self-serving bias is stronger in English than in Dutch. S4 Fig shows that the foreign language effect is practically significant–up to 1 entire point on the 7-point Likert scale–as well as statistically significant (i.e., the 95% confidence interval around the point estimate of one group does not include the point estimate of the other group).

Concerning H3 (FLA moderating the effect of language on self-serving bias), Fig 3 shows that the difference in steepness between English and Dutch is greater for higher FLA. For low FLA, the steepness is similar over the languages. That is against H3. Furthermore, H2 is not supported for any subgroup of FLA. With specifications such as those reported in S1 Fig and S3 Fig, H2 seems to be supported for the subgroup with high FLA. However, according to H3, the group with high FLA should be the least likely to see support for H2. Because H2 and H3 build on the same arguments, the arguments underlying H2 cannot be supported by a result that does not also support H3. So, because the results in those specifications are against H3, it is misleading to conclude that H2 is supported for the subgroup with high FLA.

In sum, the results support H1, are significantly the opposite of H2, and are mixed regarding H3.

### Additional analyses

Concerning the direct effect of FLA; FLA increases the self-serving bias (i.e., the relation between score and attribution to ability is steeper) in the English condition, and decreases it in the Dutch condition. However, S1 Fig and S3 Fig show the opposite result for FLA: Less self-serving bias with higher FLA (the relation between score and attribution to ability is flat). That is not because participants with high FLA are modest if they get positive feedback, but because they are more likely to admit that their low ability caused the low score. Indeed, low English ability is associated with high FLA (see also S3 Table). The plots of the observed data points in S1 Fig and S3 Fig show one reason for the sensitivity of FLA’s effect: The observations with high FLA are relatively concentrated in the easy condition, but are more spread out in the difficult condition.

Instead of the Likert scale of the attributions, one author also coded the answers to the open-ended attribution question (blind to observations’ values on the other variables). That variable takes value 1 (*n =* 27) if at least part of the answer is a causal attribution to ability, such as ‘my English vocabulary is high due to bilingual education’. The value -1 (*n* = 52) is assigned if at least part of the answer is a causal attribution to external factors, such as ‘time pressure made me nervous’. The value 0 (*n* = 41) is assigned if the answer is too distant from our four factors to be an attribution (e.g., ‘I didn’t see the words’), or if the answer contains both kind of attributions. The new variable may be informative vis-à-vis the other analyses because the answers had little straightforward connection to responses on the Likert scales. For instance, the correlation of the new variable with attribution to ability minus total other attributions is only a very low 0.13.

Multinomial logit analyses show that attribution to ability (relative to the baseline = 0) is positively related to score for participants who answered in English, and that this relationship is stronger for those with high FLA. Attribution to external factors (relative to the baseline = 0) is negatively related to score for participants who answered in English, and the steepness of that relationship does not differ depending on FLA. By contrast, for participants who answered in Dutch, attribution to ability does not seem related to score in general. However, there seems to be a positive relationship for participants with high FLA, and a negative relationship for those with low FLA. Furthermore, attribution to external factors is negatively related to score for participants with high FLA, and positively related for those with low FLA. In sum, the findings for this alternative dependent variable are in line with the main results.

We also analyzed some non-hypothesized expectations. First, we may expect that participants receiving the FLA survey before all other questions may be primed to be anxious or control-oriented, and therefore have greater self-serving bias [23]. To test that expectation, we run a model with difficulty and a dummy for getting the FLA survey before versus after the task and the interaction between those variables, with no other covariates (both variables were randomly assigned). With attribution to ability as the dependent variable, both groups react equally negatively to difficulty. That is not in line with FLA as a prime. By contrast, if the dependent variable includes subtracting the attributions to effort, task difficulty and luck (average as well as total), the participants who receive the FLA survey after the task do not react to difficulty, while the those who receive the FLA survey before the task react more self-servingly. S3 Table shows that receiving the FLA scale afterwards is not related to different levels of FLA, so that could not explain the result. In sum, there is some evidence that the FLA survey primes a control orientation.

We also investigate whether boys have greater self-serving bias than girls. The findings in our sample are in line with the meta-analysis [7]. Splitting by language also shows that the gender difference is statistically significant (>95% confidence interval) in the English condition. However, in the Dutch condition, the gender difference reverses (such that girls have greater self-serving bias), but that difference is small and statistically insignificant (i.e., within 95% confidence interval).

S3 Table shows that girls have greater FLA. That replicates a prior finding that also showed this gender difference with respect to FLA (which was mediated by personality differences) [25]. Furthermore, the negative correlation between FLA and English grade provides additional criterion validity for the FLA scale.

We treat score and the difficulty conditions as alternative measures of the valence of feedback (see, e.g., S2 Fig). Another interpretation is that a score of 10 out of 20 may be experienced as negative in the easy condition, while a score of 8 out of 20 may be experienced as positive in the difficult condition. S5 Fig reveals significant differences between the easy and difficult conditions in terms of attribution to ability and in terms of attribution to task difficulty. In terms of attribution to ability, in the easy condition, score is positively related to attribution to ability for both languages, and this relationship is steeper for participants who answer in English (in line with other results). MM-regression estimation gives equally steep positive trends for Dutch and English. In the difficult condition, score is not related to attribution to ability for participants who answered in English, but negatively so for participants who answered in Dutch. MM-regression estimation gives a neutral trend for both languages. In terms of attribution to task difficulty, in the easy condition, score has no effect for participants who answered in Dutch, and score has a negative effect for participants who answered in English. MM-regression estimation gives the same results. In the difficult condition, score is negatively related to attribution to task difficulty for participants who answered in Dutch. For participants who answered in English, score is not related to attribution to task difficulty. MM-regression estimation gives an equally steep negative trend for both Dutch and English.

In sum, the self-serving bias manifests as attribution away from ability for very low scores and as attribution away from task difficulty for very high scores. The foreign language is associated with greater self-serving bias in both situations.

## Discussion

We replicate the self-serving bias. According to theory, the self-serving bias should be greater with higher arousal [18,19], and arousal should be higher in the native vis-à-vis the foreign language [12]. However, in our sample, the self-serving bias was not greater in the native-language compared to the foreign-language condition; it was significantly less. This unexpected result may be because the foreign language does not reduce arousal for our participants. Thus, our result highlights possible boundary conditions for the arousal-reducing effect of foreign language. Specifically, the boundary condition that we explored in this study is the age of the students: 13–15 years old. Thus, all participants are early learners, which can explain why the foreign language effect is weaker [12,15]. We survey the age of acquisition of English to separate early learners from very early learners (18 students started learning English between ages 2 and 5, 69 between ages 6 and 10, and 31 at age 11 or later, for a rough average of 8.2). That additional granularity had no impact.

That weak arousal-reducing effect allows the counteracting arousal-increasing effect to dominate in our study. Our approach was to measure the extent to which that counteracting effect occurred using the foreign language anxiety (FLA) scale [25]. However, FLA may itself be plagued by counteracting effects: (1) FLA can increase the arousal caused by foreign language stimuli [21], and therefore cause a positive association between foreign language and self-serving bias (see H3); and (2) FLA is likely to be associated with low confidence, and therefore with less self-serving bias (e.g., via outcome expectancy, achievement motivation, or self-esteem [7]), especially for students who thought that the puzzle task was about their English-language skill. We observe the first effect in Fig 3, where the relation between score and attribution to ability is steeper in the English condition with high FLA. But we observe the second effect in S1 Fig and S3 Fig, where students with higher FLA are more likely to admit that a low score was due to their low ability.

**Fig 4:**
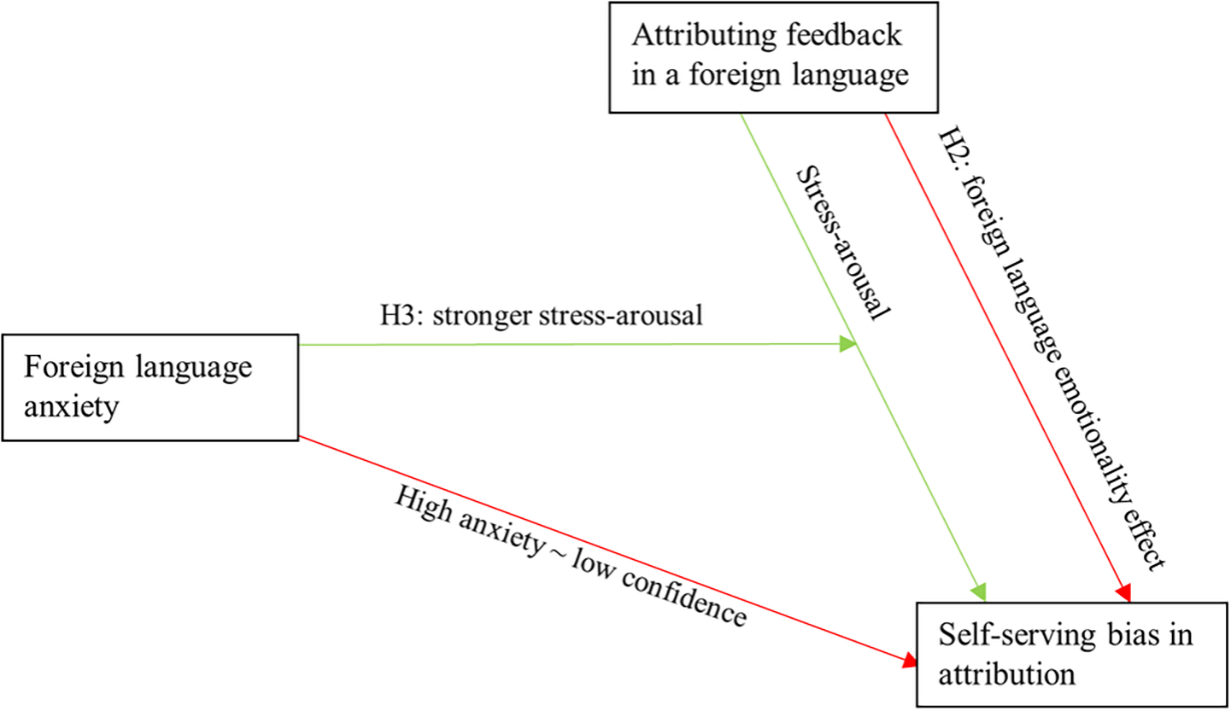
Expected conceptual model. The interaction term captures the positive covariation, such that the main effects capture only negative covariation.

**Fig 5:**
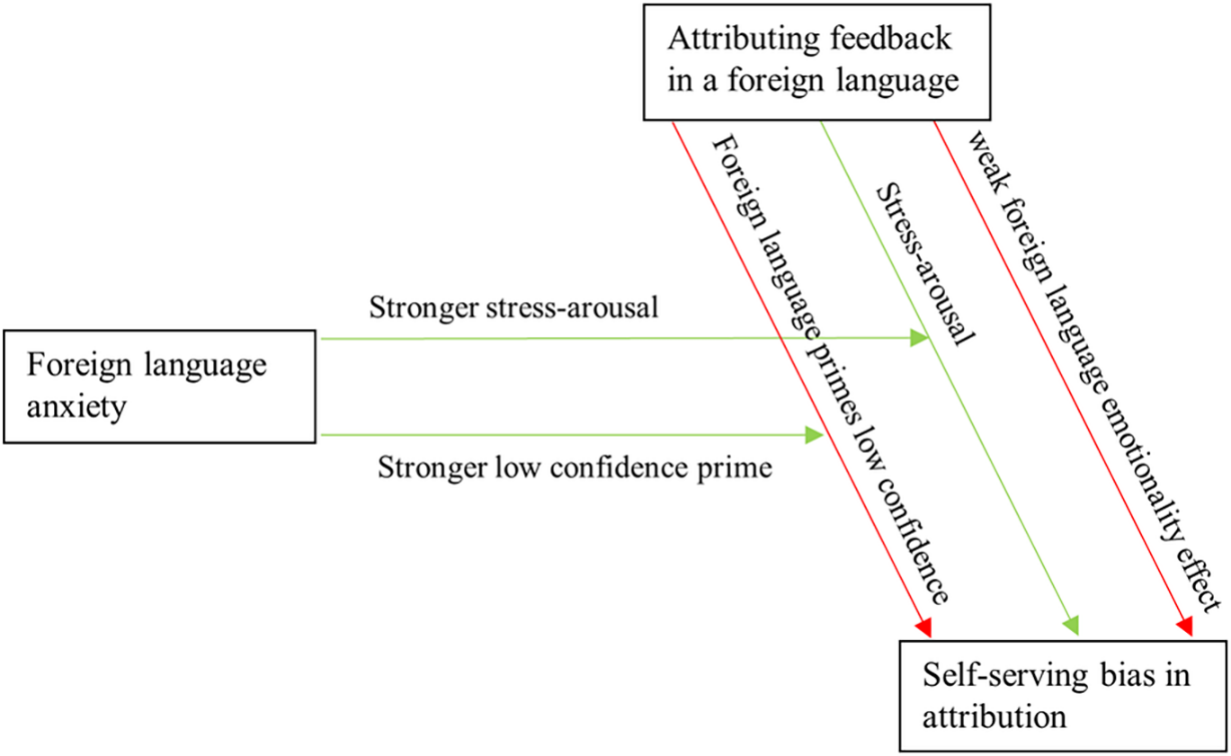
Post hoc conceptual model. The interaction term is counteracted, such that the language main effect captures both positive and negative covariation, and that negative covariation is weaker.

Fig 4 shows the mental model underlying our initial expectation to use FLA to measure the strength of the arousal-increasing effect. Fig 5 shows an ex-post mental model that explains the mixed results by appreciating how FLA is itself plagued by counteracting effects. The key difference is that FLA is directly associated with low confidence in Fig 4, while the English language of the feedback and attribution questions are needed to prime that low confidence in Fig 5. As a result, in Fig 5 the interaction between language and FLA captures a positive and a negative effect that cancel out such that the interaction effect gets assigned neither of the two effects. Instead, they are assigned to the main effect that is most closely related. Thus, the negative confidence effect gets assigned to the FLA main effect. The positive stress effect gets assigned to the language main effect, such that it counteracts H2.

### Implications for future research

FLA’s moderating effect is itself counteracted because FLA is associated with confidence and our outcome variable (i.e., self-serving bias) is also associated with confidence. So, future studies without self-serving bias as the outcome variable avoid that FLA’s moderating effect is itself counteracted. Therefore, taking FLA into account may be more successful in helping to empirically estimate the counteracting foreign-language effect in those studies.

Perhaps, future studies could capture the counteracting effect of foreign language by using variation in emotional intensity of stimuli (e.g., [14,21]). For example, such studies could randomly communicate feedback intensely (e.g., ‘You achieved an amazing score of 15 out of 20; excellent job!’ and ‘Sadly, you achieved the poor score of only 3 out of 20.’) versus neutrally (like we did in this study). The expectation would be that self-serving bias is stronger in the intense condition, and especially in the native-language-intense condition.

### Implications for teachers

Findings on a bias in attribution have implications for students’ motivation. Attributing negative feedback to situational factors is part of the process by which negative feedback reduces future motivation [9,23,24]. In other words, attributing performance to one’s own ability may increase motivation. Thus, if future studies establish further validity and generalizability of the effect of language on attribution, the findings imply that teachers can increase the motivation of students by presenting positive feedback in the foreign language, and teachers can decrease the demotivating effect of negative feedback by discussing it in the native language. Furthermore, self-serving bias to defend against negative feedback is related to self-sabotage as a defense against (future) negative feedback [23]. Therefore, if language can reduce self-serving bias, then language might also reduce self-sabotaging behavior. Moreover, choosing the language of feedback with these implications in mind can be a way to influence students’ motivation without taking away the student’s agency in the feedback attribution process, which is necessary for developing students’ self-regulation skills [27].

## Supporting information

S1 Table (DOCX)

S2 Table (DOCX)

S3 Table (DOCX)

S1 Fig (DOCX)

S2 Fig (DOCX)

S3 Fig (DOCX)

S4 Fig (DOCX)

S5 Fig (DOCX)

S1 Dataset (XLSX)
