# Supplementary material for: The foreign language effect on the self-serving bias: A field experiment in the high school classroom
Source: PLoS One. 2018 Feb 9;13(2):e0192143. doi: 10.1371/journal.pone.0192143 (PMC5806866; doi:10.1371/journal.pone.0192143)
Supplement: S1 Table — (DOCX) [file pone.0192143.s001.docx]

S6 Table. OLS and robust regression (MM) models (n=120) for 3 attribution measuresa

|  | OLS | | | Robust regression | | |
| --- | --- | --- | --- | --- | --- | --- |
|  | Ability | Ability-other(avg.) | Ability-other(total) | Ability | Ability-other(avg.) | Ability-other(total) |
| Score | -0.18 | -0.09 | 0.08 | 0.02 | -0.06 | 0.22 |
|  | (0.14) | (0.15) | (0.28) | (0.08) | (0.13) | (0.38) |
| English answer | -2.77 | -3.62 | -5.34 | -3.27 | -1.96 | -1.94 |
|  | (1.80) | (2.31) | (4.15) | (1.40) | (1.88) | (8.11) |
| English-Score Interaction | 0.33 | 0.32 | 0.30 | 0.39 | 0.20 | -0.02 |
|  | (0.19) | (0.21) | (0.38) | (0.12) | (0.16) | (0.61) |
| FLA | -0.49 | -0.71 | -1.14 | 0.52 | -0.64 | -0.13 |
|  | (0.43) | (0.56) | (1.05) | (0.22) | (0.76) | (2.02) |
| Score-FLA interaction | 0.07 | 0.06 | 0.02 | -0.00 | 0.04 | -0.08 |
|  | (0.04) | (0.05) | (0.10) | (0.02) | (0.06) | (0.16) |
| English-FLA interaction | 0.97 | 1.11 | 1.40 | 0.68 | 0.48 | -0.31 |
|  | (0.60) | (0.80) | (1.45) | (0.40) | (0.76) | (2.68) |
| English-Score-FLA interaction | -0.10 | -0.09 | -0.06 | -0.09 | -0.05 | 0.10 |
|  | (0.06) | (0.07) | (0.13) | (0.03) | (0.06) | (0.21) |
| Female | -0.45 | -0.33 | -0.10 | -0.21 | -0.56 | -0.23 |
|  | (0.30) | (0.34) | (0.67) | (0.27) | (0.52) | (1.05) |
| 3^rd^ Year | 0.06 | 0.00 | -0.12 | 0.45 | 0.60 | 0.86 |
|  | (0.30) | (0.36) | (0.70) | (0.30) | (0.58) | (1.15) |
| English grade | -0.11 | -0.05 | 0.07 | 0.44 | 0.06 | -0.07 |
|  | (0.20) | (0.23) | (0.46) | (0.17) | (0.31) | (0.76) |
| Constant | 6.09 | 2.44 | -4.86 | -0.55 | 1.32 | -5.84 |
|  | (1.97) | (2.43) | (4.86) | (1.57) | (3.54) | (8.78) |
| *R*^2^ | 0.09 | 0.11 | 0.12 |  |  |  |

^a^ Robust standard errors in parentheses.

The statistical significance of the regression coefficients is difficult to interpret. For example, a test for statistical significance compares the coefficient of ‘English answer’ (e.g., -2.77 to 0). However, the effect of that variable varies depending on ‘Score’ and ‘FLA’, and the coefficient represents the effect of ‘English answer’ if Score=0 and FLA=0. Thus, whether -2.77 differs statistically significantly from 0 is uninformative about the complete effect of ‘English answer’. In fact, FLA=0 is not even possible in the data, so the coefficient of -2.77 and its statistical significance are meaningless. For score-FLA interaction, the coefficient (e.g., 0.07 in the first model) is the coefficient if ‘English answer’=0 (i.e., the answer is in Dutch). However, both that coefficient and the coefficient on the triple interaction are compared to 0 rather than compared to each other. So, the tables are unhelpful in telling us if ‘Score-FLA interaction’ differs significantly between the languages. See S4 Fig for a closer look at statistical significance.

Fig 2 of the paper is based on a model like Model 3 except for using answer language instead of question language. S2B Fig shows that that difference is minor.
