# Supplementary material for: The foreign language effect on the self-serving bias: A field experiment in the high school classroom
Source: PLoS One. 2018 Feb 9;13(2):e0192143. doi: 10.1371/journal.pone.0192143 (PMC5806866; doi:10.1371/journal.pone.0192143)
Supplement: S2 Table — (DOCX) [file pone.0192143.s002.docx]

S7 Table. Full correlation table.

Mean S.D. Min Max (1) (2) (3) (4) (5) (6) (7) (8) (9) (10) (11) (12) (13) (14)

(1)Ability Attribution 4.05 1.55 1 7

(2)Ability-average other 0.40 1.81 -5 5 0.78

(3)Ability-total other -6.91 3.57 -17 2 0.33 0.84

(4)Difficult condition 0.48 0.50 0 1 -0.16 -0.23 -0.21

(5)Score 7.72 5.27 0 17 0.17 0.27 0.27 -0.89

(6)Dutch condition 0.53 0.50 0 1 -0.08 0.02 0.09 0.05 0.02

(7)English answer 0.53 0.50 0 1 0.05 -0.00 -0.05 -0.06 0.01 -0.86

(8)Interact(5)x(7) 4.14 5.40 0 17 0.13 0.17 0.15 -0.48 0.50 -0.61 0.72

(9)FLA 2.98 1.07 0.9 6.5 0.09 -0.02 -0.12 -0.07 0.09 0.01 -0.04 -0.04

(10)Interact(5)x(9) 23.48 19.51 0 75.6 0.18 0.20 0.15 -0.74 0.85 0.02 -0.05 0.36 0.54

(11)Interact(7)x(9) 1.57 1.68 0 5.5 0.08 0.01 -0.06 -0.02 -0.00 -0.78 0.88 0.62 0.34 0.12

(12)Interact(5)x(7)x(9) 12.07 17.71 0 75.6 0.12 0.15 0.11 -0.41 0.44 -0.59 0.64 0.89 0.26 0.51 0.74

(13)Female=1 0.62 0.49 0 1 -0.12 -0.10 -0.06 0.08 -0.04 0.04 0.02 -0.02 0.26 0.11 0.11 0.07

(14)Year(3rd=1) 0.42 0.50 0 1 0.03 0.04 0.03 -0.01 0.02 0.09 -0.06 0.02 -0.18 -0.05 -0.09 -0.00 -0.13

(15)English grade 7.13 0.79 6 8.5 -0.10 -0.02 0.06 0.14 -0.03 0.17 -0.07 0.03 -0.16 -0.12 -0.10 -0.02 0.20 -0.13
