# Supplementary material for: The foreign language effect on the self-serving bias: A field experiment in the high school classroom
Source: PLoS One. 2018 Feb 9;13(2):e0192143. doi: 10.1371/journal.pone.0192143 (PMC5806866; doi:10.1371/journal.pone.0192143)
Supplement: S3 Table — (DOCX) [file pone.0192143.s003.docx]

**S8 Table. OLS regression on foreign language anxiety (FLA).**

|  | FLA | FLA |
| --- | --- | --- |
| Female | 0.63 | 0.63 |
|  | (0.17) | (0.18) |
| 3^rd^ year | -0.36 | -0.37 |
|  | (0.18) | (0.19) |
| English Grade | -0.33 | -0.33 |
|  | (0.11) | (0.11) |
| FLA survey afterwards^a^ | -0.03 | -0.05 |
|  | (0.18) | (0.18) |
| Difficult condition |  | -0.07 |
|  |  | (0.28) |
| Dutch condition |  | 0.19 |
|  |  | (0.27) |
| Difficult-Dutch interaction |  | -0.12 |
|  |  | (0.37) |
| Constant | 5.09 | 5.07 |
|  | (0.83) | (0.84) |
| *R*^2^ | 0.15 | 0.15 |

^a^ Subjects were randomly assigned to receive the FLA scale before or after all other questions.
