# Supplementary material for: The foreign language effect on the self-serving bias: A field experiment in the high school classroom
Source: PLoS One. 2018 Feb 9;13(2):e0192143. doi: 10.1371/journal.pone.0192143 (PMC5806866; doi:10.1371/journal.pone.0192143)
Supplement: S1 Fig — (DOCX) [file pone.0192143.s004.docx]

S1A Fig. Observed attribution to ability over the experimental conditions.

S1B Fig. OLS prediction of attribution to ability over the experimental conditions.

The prediction in Figure A1b is based on an OLS model with the following covariates: difficulty condition, language condition, FLA and its interaction with the conditions separately and a three-way interaction, and control variables for gender, current average English grade, and whether the participant was in the 2^nd^ or 3^rd^ year. The graphs use Stata’s jitter option, which adds a bit of random noise to separate different observations with the same location, so that the graphs can show the density of observations at different locations.
