# Supplementary material for: The foreign language effect on the self-serving bias: A field experiment in the high school classroom
Source: PLoS One. 2018 Feb 9;13(2):e0192143. doi: 10.1371/journal.pone.0192143 (PMC5806866; doi:10.1371/journal.pone.0192143)
Supplement: S2 Fig — (DOCX) [file pone.0192143.s005.docx]

Figure A2. Experimental conditions over more nuanced alternative measurements. (A) Randomly assigned task difficulty versus actual score. (B) Randomly assigned question language versus participant’s answer’s language. (C) Likert scale attributions versus coded open-ended attributions by experimental conditions.

Score is higher in the easy condition than in the difficult condition, so the manipulation worked: the easy-difficult condition randomly assigned subjects to high-low score. More details can be found in Figure A5.

Most of the circles are red and most of the diamonds are blue, so the manipulation worked, and the language of the answers is a useful alternative measure to the language of the experimental condition. Participants who answered in English to their Dutch question had somewhat lower foreign language anxiety than other participants. The experimental condition is about the language in which feedback is received. The language of the answer is about the language in which participants communicate their attribution. In between those steps is the language in which attribution is processed. If the languages of the question and answer do not match, then our theory cannot work.

There is little correspondence between the responses to the Likert scale and the coded responses to the open-ended attribution question. The correlation between the coded response and the Likert scale attribution to ability is 0.08, and for the Likert attribution to task difficulty it is -0.08.

There are more blue diamonds in the easy conditions (reflecting findings supporting for H1), and especially in the easy-English condition (reflecting results opposing H2).
