# Supplementary material for: The foreign language effect on the self-serving bias: A field experiment in the high school classroom
Source: PLoS One. 2018 Feb 9;13(2):e0192143. doi: 10.1371/journal.pone.0192143 (PMC5806866; doi:10.1371/journal.pone.0192143)
Supplement: S3 Fig — (DOCX) [file pone.0192143.s006.docx]

S3A Fig. Observed attribution to ability by score and language of participant’s answer.

S3B Fig. OLS prediction of attribution to ability by score and answer language.

S3C Fig. A model like A3b but without FLA for participants that answered in Dutch.

S3D Fig. MM regression prediction of attribution to ability over score and answer language.

The predictions in S3B and S3D Figs are based on Models 1 and 4 in S6 Table, respectively. Participants reacted more self-servingly to feedback when they received that feedback and communicated their attribution in the foreign language (i.e. English). In S3B and S3D Figs, the variables are the same, but the estimation method is different.

S3B Fig is like Fig 2 except for subtracting total other attributions (in addition to the small difference of using language condition instead of answer language). Comparing the two figures suggests that the effects of language and score are robust to different attribution measures. However, the effect of FLA is not robust: While Fig 2 suggests that higher FLA is associated with stronger self-serving bias (i.e., steeper increase of attribution over score) in English and weaker self-serving bias in Dutch, S3B Fig suggests that higher FLA is associated with weaker self-serving bias in English and stronger self-serving bias in Dutch.

Because OLS minimizes the sum of *squared* errors, large errors have large ‘weight’ in the estimation and small errors have small ‘weight’. Thus, outliers become more influential. Robust MM regression puts a small weight on observations with large error (Starbuck, 2015). Comparing to S3A Fig shows how the different estimation methods differently process the raw observations.
