# Supplementary material for: The foreign language effect on the self-serving bias: A field experiment in the high school classroom
Source: PLoS One. 2018 Feb 9;13(2):e0192143. doi: 10.1371/journal.pone.0192143 (PMC5806866; doi:10.1371/journal.pone.0192143)
Supplement: S4 Fig — (DOCX) [file pone.0192143.s007.docx]

S4A Fig. Marginal effects of Dutch on attribution to ability (with 95% CI).

S4A Fig graphs how much attribution to ability changes if we move from English to Dutch. The predictions are based on the same model as S1B Fig. By contrast, the models in S6 Table show little statistical significance, because those models have the more granular ‘score’ variable instead of the dichotomous ‘difficulty condition’ variable. Increasing ‘difficulty condition’ by 1 is like increasing ‘score’ by 10. In line with this, the effect size of ‘difficulty condition’ tends to be around 10 times greater than the effect size of ‘score’. However, the standard error tends to be only around 5 times larger. That accounts for the statistical significance.

In the easy condition, and for FLA=2 and FLA=5, the 95% confidence interval of the marginal effect of Dutch does not include 0 and does not include the point-estimate of the difficult condition. Thus, in the easy condition, students with FLA=2 are statistically significantly more modest in Dutch than they are in English, and significantly more modest in Dutch than they are in the difficult condition (in Dutch). Also, in the easy condition, students with FLA=5 are statistically significantly more boastful in Dutch than they are in English, and significantly more boastful in Dutch than they are in the difficult condition (in Dutch). In other words, students who feel anxious in English are more modest about the role of their ability in getting a good score if they get asked the question in English as opposed to Dutch.

S4B and S4C Figs show how these results are sensitive to specifications. For example, S4B Fig graphs the same model from the perspective of the difficult condition. All confidence intervals include 0, but for FLA=2, there is a significant difference between Dutch and English. S4C Fig is the same as S4A Fig except the dependent variable is attribution to ability minus total other attributions. Everything is insignificant. That is striking given that, in Fig 2 (which has the same dependent variable as S4C Fig), the scatters show a starkly different steepness between the languages.

S4B Fig. Marginal effects of Difficult on attribution to ability (with 95% CI).

S4C Fig. Marginal effect of Dutch on attribution to ability minus total other attributions.
