# Supplementary material for: The foreign language effect on the self-serving bias: A field experiment in the high school classroom
Source: PLoS One. 2018 Feb 9;13(2):e0192143. doi: 10.1371/journal.pone.0192143 (PMC5806866; doi:10.1371/journal.pone.0192143)
Supplement: S5 Fig — (DOCX) [file pone.0192143.s008.docx]

S5A Fig. Observed attribution to ability by difficulty condition.

S5B Fig. OLS prediction of attribution to ability by difficulty condition.

S5C Fig. Observed attribution to task difficulty by difficulty condition.

S5D Fig. OLS prediction of attribution to task difficulty by difficulty condition

The OLS models exaggerate trends that seem small in the plots of observed values. MM-regression predictions give similar plots, except for smaller differences between the languages.
